# Lidocaine effects on neutrophil extracellular trapping and angiogenesis biomarkers in postoperative breast cancer patients with different anesthesia methods: a prospective, randomized trial

**DOI:** 10.1186/s12871-024-02540-7

**Published:** 2024-04-27

**Authors:** Wenjuan Zhang, Jiao Liu, Xiaohui Li, Zhixia Bai, Yan Sun, Xuexin Chen

**Affiliations:** 1 School of Clinical Medicine, Ningxia Medical University, No.692 Shengli South Street Xingqing District, Yinchuan, 750004 Ningxia China; 2 Department of Anaesthesia and Perioperative Medicine, Cancer Hospital, General Hospital of Ningxia Medical University, No.804 Shengli South Street Xingqing District, Yinchuan, 750004 Ningxia China

**Keywords:** Breast cancer, Lidocaine, Neutrophil extracellular trapping, Angiogenesis, Propofol, Sevoflurane, Recurrence

## Abstract

**Background:**

Anesthesia techniques and drug selection may influence tumor recurrence and metastasis. Neutrophil extracellular trapping (NETosis), an immunological process, has been linked to an increased susceptibility to metastasis in individuals with tumors. Furthermore, recurrence may be associated with vascular endothelial growth factor A (VEGF-A), a mediator of angiogenesis. This study investigates the impact of lidocaine (combined with sevoflurane or propofol anesthesia ) during breast cancer surgery inhibits the expression of biomarkers associated with metastasis and recurrence (specifically H3Cit, NE, MPO, MMP-9 and VEGF-A).

**Methods:**

We randomly assigned 120 women undergoing primary or invasive breast tumor resection to receive one of four anesthetics: sevoflurane (S), sevoflurane plus i.v. lidocaine (SL), propofol (P), and propofol plus i.v. lidocaine (PL). Blood samples were collected before induction and 3 h after the operation. Biomarkers associated with NETosis (citrullinated histone H3 [H3Cit], myeloperoxidase [MPO], and neutrophil elastase [NE]) and angiogenesis were quantified using enzyme-linked immunosorbent assays.

**Results:**

Patient and breast tumor characteristics, along with perioperative management, did not differ between study groups. In intra-group comparisons, S and P groups demonstrated a statistically significant increase in post-operative MPO (S group: 10.39[6.89–17.22] vs. 14.31[8.55–20.87] ng ml-1, *P* = 0.032; P group: 9.45[6.73–17.37] vs. 14.34[9.87–19.75] ng ml-1, *P* = 0.035)and NE(S group: 182.70[85.66-285.85] vs. 226.20[91.85-391.65] ng ml-1, *P* = 0.045; P group: 154.22[97.31–325.30] vs. 308.66[132.36-483.57] ng ml-1, *P* = 0.037) concentrations compared to pre-operative measurements, whereas SL and PL groups did not display a similar increase. H3Cit, MMP-9, and VEGF-A concentrations were not significantly influenced by the anesthesia techniques and drugs.

**Conclusions:**

Regardless of the specific technique employed for general anesthesia, there was no increase in the postoperative serum concentrations of MPO and NE after perioperative lidocaine infusion compared to preoperative serum concentrations. This supports the hypothesis that intravenous lidocaine during cancer surgery aimed at achieving a cure may potentially decrease the likelihood of recurrence. Further interpretation and discussion of clinical implications are warranted, emphasizing the significance of these findings in the context of cancer surgery and recurrence prevention.

**Clinical trial registration:**

ChiCTR2300068563.

## Background

Cancer, a global health challenge, ranks as the leading or second-leading cause of death worldwide and holds the top position in China. Notably, female breast cancer has surpassed lung cancer as the most frequently diagnosed cancer globally and remains the primary cause of cancer-related deaths among women [1]. The predominant management approach for breast cancer involves surgical resection. However, this procedure induces a stress response, leading to the release of various pro-inflammatory cytokines and molecules into the bloodstream. These substances have the potential to compromise cell-mediated immunity, creating an environment conducive to the survival and growth of residual tumor cells, thereby accelerating tumor cell proliferation and facilitating premature metastasis [2]. 

To enhance the cure and survival rates for breast cancer, it is imperative to reduce local recurrence and metastasis following surgery. The interaction between anesthesia techniques, drug selection, and the cellular immune system may be factors that influence tumor recurrence and metastasis [3]. Propofol-based total intravenous anesthesia (TIVA) has demonstrated enhanced recurrence-free survival and overall survival compared to inhalation anesthesia in both animal models and retrospective clinical studies [4, 5]. Nevertheless, a recent CAN study comprising 1764 breast cancer patients showed no difference in overall survival between general anesthesia with propofol or sevoflurane for breast cancer surgery [6]. Notably, local anesthetics, particularly the amide local anesthetic lidocaine, exhibit significant non-local anesthetic effects, including potent analgesic and anti-inflammatory effects as a systemic intravenous infusion [7]. Recent studies have unveiled lidocaine’s distinct anticancer properties, such as inducing tumor cell apoptosis and regulating tumor cell biology [8]. 

Neutrophil extracellular trapping (NETosis), a novel biomarker of metastasis risk, has been identified as a critical immune response in cancer progression and metastasis [9, 10]. NETosis involves the release of neutrophil extracellular traps (NETs) when neutrophils come into contact with tumor cells [11]. This cascade of events releases various contents, including histones, granule proteases, and cytosolic proteins, into the circulation [12, 13]. High levels of NETosis in tumor samples are associated with poor prognosis, and increased NETosis is linked to breast cancer recurrence and metastasis [9]. Additionally, vascular endothelial growth factor A (VEGF-A) plays a crucial role in tumor angiogenesis, influencing tumor growth and serving as an unfavorable prognostic indicator for tumor-free survival [14, 15]. 

Lidocaine’s potential to reduce the expression of NETosis-specific markers and VEGF-A through its specific anticancer effects suggests a promising avenue for intervention. While the impact of propofol-TIVA and sevoflurane anesthesia on these outcomes remains uncertain, there is a growing belief that sevoflurane may elevate the risk of metastasis and recurrence. Therefore, we hypothesized that lidocaine (combined with sevoflurane or propofol anesthesia) during breast cancer surgery inhibits the expression of biomarkers associated with metastasis and recurrence (specifically H3Cit, NE, MPO, MMP-9 and VEGF-A).

## Materials and methods

### Study design and participants

This study employed a prospective, controlled, parallel-group clinical trial design with equal randomization, conducted at Ningxia Medical University General Hospital affiliated Cancer Hospital. The study received ethical approval from the Ethic Committee of Ningxia Medical University General Hospital (approval number: KYLL-2023-0045) and was registered a priori with the Chinese Clinical Trial Registry (www.chictr.org.cn, ChiCTR2300068563; 23/02/2023). The enrollment period spanned from February 2023 to October 2023. We used the CONSORT checklist when writing our report [16].

Adhering to Good Clinical Practice guidelines and the Declaration of Helsinki, written informed consent was obtained from participating patients or authorized surrogates. The trial considered ethical principles to protect patient rights and well-being.

Inclusion criteria encompassed patients aged 18–70 years, ASA physical status 1–3, with primary or invasive breast cancer without distant metastasis (stage 0 to III), and with or without axillary lymph node dissection. Exclusion criteria included allergies to study substances, diabetes, coronary heart disease, chronic inflammatory diseases, previous surgical history of breast cancer (except diagnostic biopsy), neuropsychiatric diseases hindering informed consent, incapacity to understand the study protocol or refusal to participate, and regular usage of corticosteroids or anti-inflammatory drugs.

### Randomization and masking

Patients in this study were allocated to one of four groups using a computer-generated randomization process with a ratio of 1:1:1:1. To ensure blinding, group assignments and patient study numbers were concealed within opaque, sealed envelopes. These envelopes were opened only after patients signed a written informed consent form preoperatively.

The experimental groups included sevoflurane anesthesia (S), sevoflurane anesthesia plus intravenous lidocaine (SL), propofol total intravenous anesthesia (P), and propofol total intravenous anesthesia plus intravenous lidocaine (PL). The selection of these anesthesia methods was based on their relevance to our research question and their common use in breast cancer surgery.

For safety reasons, the attending anesthesiologist used non-blind criteria throughout the lidocaine infusion. To maintain the integrity of the blinding process, researchers involved in postoperative follow-up, blood collection, laboratory testing, data analysis, and interpretation remained unaware of the grouping.

Non-lidocaine groups received a placebo infusion of saline.

### Procedures

Patients in this study underwent routine fasting for 8 h and were restricted from drinking 4 h prior to surgery. Additionally, 30 min before anesthesia, patients received an intramuscular injection of pentylenetetrazol hydrochloride (1 mg). This pre-anesthetic medication aims to inhibit salivary gland and airway gland secretion.

During anesthesia induction, all patients across the four groups (S, SL, P, PL) received a standardized regimen consisting of intravenous midazolam (0.05 mg kg^−1^), sufentanil (0.3 µg kg^−1^), etomidate (0.3 mg kg^−1^), and rocuronium (0.6 mg kg^−1^). Following laryngeal mask placement, mechanical ventilation was employed to maintain the end-expiratory carbon dioxide concentration at 35–45 mmHg with a 50/50 mixture of O2/air at a flow rate of 2 L/min.

For the propofol total intravenous anesthesia (TIVA) groups (P and PL), anesthesia maintenance involved a constant infusion of propofol (3–5 mg kg^−1^ h^−1^) and remifentanil (0.3 µg kg^−1^ min^−1^) to sustain a bispectral index (BIS) within the range of 40–60, ensuring optimal analgesia.

In the sevoflurane groups (S and SL), anesthesia maintenance included continuous inhalation of 1-3% sevoflurane (1-1.5 MAC) to maintain BIS values between 40 and 60. Simultaneously, remifentanil was continuously administered at 0.3 µg kg-1 min-1 for intraoperative analgesia. Intraoperatively, mean arterial pressure was maintained within 20% of the basal value, and the use of vasoactive drugs (e.g., ephedrine) was determined by the attending anesthesiologist.

For the lidocaine groups (PL and SL), a loading dose of 1% lidocaine (1.5 mg kg^−1^) was administered during induction, followed by a continuous infusion of lidocaine at 2 mg kg^−1^ h^−1^ throughout the procedure. Groups P and S received an equal volume of saline instead of lidocaine.

Although the dosing protocols maintained plasma concentrations below toxic levels, a 20% lipid emulsion was prepared in the operating room as a safety precaution. Anesthesiologists were informed of its location. Midazolam (0.05 mg kg^−1^) was administered for local anesthetic poisoning seizures. If symptoms persisted or the patient’s condition was unstable, a rapid intravenous loading dose of 20% lipid emulsion (1.5 ml kg^−1^) was given, followed by continuous micropump infusion at a rate of 0.25 ml kg^−1^ min^−1^. The loading dose could be repeated (up to three times), and the infusion rate could be increased, not exceeding 0.5 ml kg^−1^ min^−1^.

At the end of surgery, neuromuscular antagonism was achieved through the administration of neostigmine (1 mg) and atropine (0.5 mg). Postoperative analgesia included the initial choice of acetaminophen (1 g), administered when patients reported pain in the post-anesthesia care unit and ward.

### Neutrophil extracellular traps and VEGF assays

The primary outcomes encompassed serum concentrations of H3Cit, MPO, NE, MMP-9, and VEGF-A. Venous blood samples (5 ml) were collected from each patient before anesthesia induction and 3 h post-operation [17], using serum separator tubes (KWS, Hebei, China). This collection, performed from a different venous access site and then used for drug administration, aimed to collect blood samples during the peak release of neutrophil extracellular trapping markers and to reduce instances of patient culling resulting from self-administered oral medication. Post-collection, blood was centrifuged at 3000 times per minute for 20 min at 4–6 °C within 3 h. The resulting supernatants were transferred into 2 ml aliquots at -80 °C for subsequent enzyme-linked immunosorbent assay (ELISA) analysis.

ELISA measurements were conducted using commercially available kits for H3Cit, MPO, NE, MMP-9, and VEGF-A (Jianglai Bio and Boster, China) following the manufacturer’s instructions.

The sandwich ELISA technique determined MPO levels, utilizing 96-well plates pre-coated with anti-MPO antibodies. A biotin-conjugated anti-MPO antibody and horseradish peroxidase (HRP)-streptavidin conjugate (SABC solution) facilitated the detection. The HRP substrate, 3,3’,5,5’-Tetramethylbenzidine (TMB), initiated the color change. In brief, serum samples were thawed and diluted (1:10) with sample dilution buffer. Test sample dilutions (0.1 ml aliquots) were added to wells, sealed, and incubated at 37 °C. Subsequent steps included the addition of a biotin-conjugated detection antibody, SABC working solution, and TMB substrate. Absorbance at 450 nm was measured using the ELISA Thermo Scientific™ Multiskan™ FC Enzyme Labeler.

The serum concentration of each factor was determined from standard and control samples, with curves drawn on coordinate paper. For accuracy, each factor underwent a repeat measurement. Inter-batch and intra-assay coefficients of variation were assessed, with values meeting the manufacturer’s specifications.

Perioperative data, including patient and breast tumor characteristics, anesthetic and surgical factors, and intraoperative details, were also collected. This additional information provides a comprehensive understanding of the study context. These meticulous procedures ensure the robustness of our data collection and analysis, contributing to the reliability of our study outcomes.

### Sample size and statistical analysis

In anticipation of a scientifically significant reduction of 2.0 ng ml-1, approximately 20% lower than the typical serum estimations of NETosis MPO values (10–15 ng ml-1 with a standard deviation of 3 ng ml-1), we conducted a power analysis. Assuming a type I error of 0.05 and a type II error of 0.1, a sample size of *n* = 25 patients per group would yield 90% power to detect this anticipated difference. To account for potential missing data, we enrolled *n* = 30 patients in each group.

For statistical analysis, GraphPad Prism TM v9 was employed. Data normality and homogeneity were assessed. Normally distributed and homogenous data were subjected to analysis of variance (ANOVA) with post hoc Bonferroni correction for intergroup comparisons. The choice of ANOVA aimed to capture differences between independent groups effectively.

Within-group differences in serum marker values before and after anesthesia and surgery for normally distributed data were assessed using paired Student’s t-tests. Skewed or uneven variances data were analyzed using the Kruskal-Wallis H test with post hoc Bonferroni correction for group comparisons, while within-group differences were assessed using the Wilcoxon test.

Categorical variables underwent analysis using chi-square tests, continuity correction chi-square tests, or Fisher exact tests, depending on the nature of the data. Data presentation followed standard conventions: mean (standard deviation), median (25–75% interquartile range), or n (%).

GraphPad Prism TM v9 was chosen for its suitability for biomedical research and its user-friendly interface. A threshold of *P* < 0.05 was considered statistically significant. This robust statistical approach ensures a comprehensive analysis of our data, aligning with the scientific rigor required for meaningful interpretation and drawing valid conclusions from our study.

## Results

**Fig. 1:**
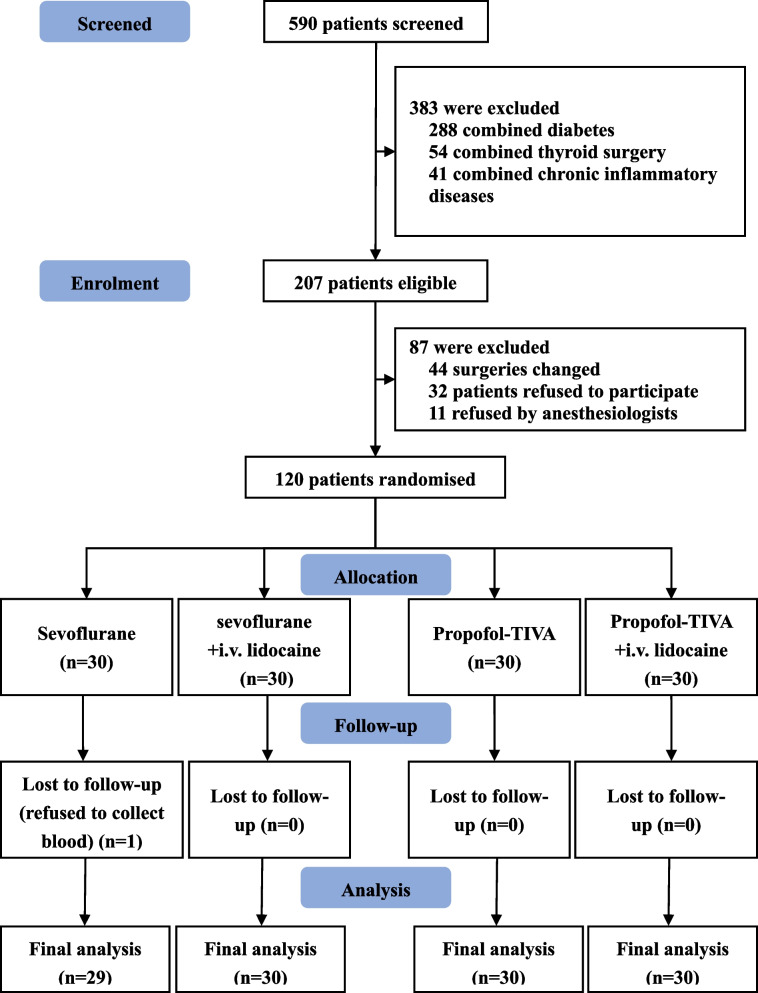
CONSORT flow diagram of participants allocation

Between February 1, 2023, and October 31, 2023, a total of 120 patients were enrolled in our study. These participants were randomly assigned to one of the four study groups: sevoflurane anesthesia (S), sevoflurane anesthesia plus intravenous lidocaine (SL), propofol-TIVA (P), and propofol-TIVA plus intravenous lidocaine (PL). Unfortunately, one patient from the sevoflurane anesthesia group (S) was lost to follow-up due to their refusal to participate in the postoperative blood collection process (see Fig. 1).

The baseline characteristics of subjects, including ASA physical status, prevalence of hypertensive patients, tumor TNM stage, type of surgery, postoperative pathologic staging, molecular typing, and duration of anesthesia and surgery, were comparable among the study groups (see Tables 1 and 2 for specific data ranges). The distribution of patients who received preoperative chemotherapy was similar across all four groups (Table 1). Additionally, intraoperative drug consumption exhibited no significant differences among the study groups (Table 2).

**Table 1:** Patient and breast tumour characteristics

Parameter	Group S	Group SL	Group P	Group PL
	Sevoflurane	Sevoflurane + lidocaine	Propofol-TIVA	Propofol-TIVA + lidocaine
	(*n* = 29)	(*n* = 30)	(*n* = 30)	(*n *= 30)
Age (yr)	50.3(9.3)	53.6(10.5)	51.1(7.8)	50.5(8.1)
BMI (kg m2)	23.3(3.1)	23.5(2.7)	24.5(2.9)	23.8(2.4)
ASA physical status (n,%)				
I	8(28)	7(23)	5(17)	6(20)
II	21(72)	23(77)	25(83)	24(80)
Hypertensive (n,%)	2(7)	6(20)	5(17)	3(10)
Preoperative NACT (n,%)	3(10)	2(7)	1(3)	1(3)
Tumour site (n,%)				
Left	13(45)	11(37)	15(50)	19(63)
Right	16(55)	19(63)	15(50)	11(37)
TNM classification				
Pathology stage, tumour (n,%)				
Tis	1(3)	2(7)	2(7)	3(10)
T0	0	1(3)	0	0
T1	18(62)	17(57)	20(67)	19(63)
T2	10(35)	10(33)	8(27)	8(27)
Pathology stage, nodes (n,%)				
N0	18(62)	20(67)	16(53)	23(77)
N1	7(24)	5(17)	10(33)	4(13)
N2	2(7)	4(13)	2(7)	2(7)
N3	2(7)	1(3)	2(7)	1(3)
Pathology stage, metastasis (n,%)				
M0	29(100)	30(100)	30(100)	30(100)
Tumour TNM stage (n,%)				
0	1(3)	2(7)	2(7)	3(10)
I	12(41)	12(40)	11(37)	17(57)
II	12(41)	11(37)	13(43)	7(23)
III	4(14)	5(17)	4(13)	3(10)

All data shown are mean (standard deviation) or n (%)

*TIVA* Total Intravenous Anesthesia, *BMI* Body Mass Index, *ASA* American Society of Anesthesiologists, *NACT* New Adjuvant Chemotherapy Treatment, *TNM* Tumor-Node-Metastasis, *Tis* Tumor in situ

**Table 2:** Surgical and anaesthesia characteristics

Parameter	Group S	Group SL	Group P	Group PL
	Sevoflurane	Sevoflurane +lidocaine	Propofol-TIVA	Propofol-TIVA + lidocaine
	(*n* = 29)	(*n* = 30)	(*n* = 30)	(*n* = 30)
Type of surgical intervention (n,%)				
Wide local excision with node dissection	8(28)	6(20)	6(20)	7(23)
Modified radical mastectomy	12(41)	19(63)	20(67)	12(40)
Radical mastectomy	9(31)	4(13)	4(13)	10(33)
Simple mastectomy	0	1(3)	0	1(3)
Postoperative pathologic staging (n,%)				
Invasive breast carcinoma NST	27(93)	28(93)	25(83)	25(83)
Invasive lobular carcinoma	1(3)	0	1(3)	1(3)
Adenoid cystic carcinoma	0	0	0	1(3)
In situ ductal carcinoma	1(3)	1(3)	3(10)	3(10)
Mucinous adenocarcinoma with invasive breast carcinoma NST	0	0	1(3)	0
Intraductal papillary carcinoma	0	1(3)	0	0
Postoperative molecular typing (n,%)				
Luminal A	4(14)	2(7)	10(33)	5(17)
Luminal B(HER2 negative)	17(59)	16(53)	9(30)	15(50)
Luminal B(HER2 positive)	0	3(10)	2(7)	3(10)
Erb-B2 overexpression	1(3)	2(7)	4(13)	2(7)
Basal-like	6(21)	5(17)	4(13)	5(17)
No available data	1(3)	2(7)	1(3)	0
Duration of surgery (min)	82.55(20.41)	84.43(21.74)	76.83(28.53)	77.57(26.63)
Duration of anaesthesia (min)	104.86(24.78)	105.07(24.05)	96.10(30.57)	97.67(30.50)
Intraoperative Midazolam (mg)	3.03(0.48)	2.97(0.32)	3.03(0.37)	3.03(0.32)
Intraoperative Sufentanil (ug)	18.15(2.90)	17.80(1.95)	18.17(2.21)	18.16(1.93)
Intraoperative Remifentanil (mg)	0.99 (0.28)	0.96 (0.27)	1.10(0.45)	0.95 (0.35)
Intraoperative Etomidate (mg)	18.15(2.90)	17.80(1.95)	18.17(2.21)	18.16(1.93)
Intraoperative Rocuronium (mg)	43.84(7.39)	40.05(6.49)	41.54(7.00)	39.65(6.43)
Intraoperative HR	66.03(11.20)	64.23(11.22)	63.83(6.83)	64.03(9.64)
Intraoperative MAP (mmHg)	70.17(10.48)	71.83(11.47)	76.53(13.87)	73.37(10.63)

All data shown are mean (standard deviation) or n (%)

*NST *No special type, *HER2* Human epidermal growth factor receptor 2, *HR* Heart rate, *MAP* Mean arterial pressure

**Fig. 2:**
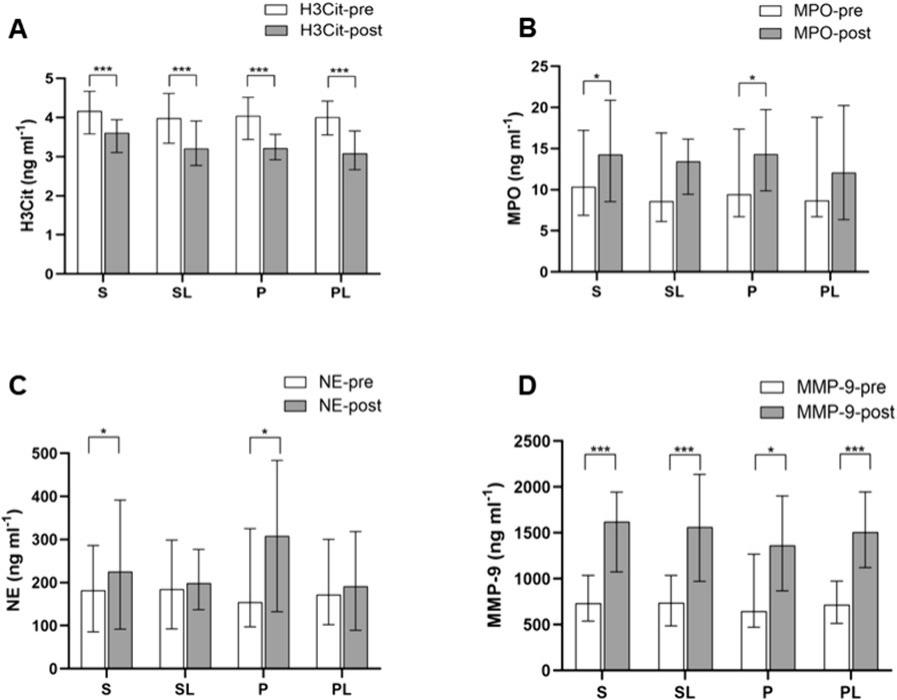
**A** Citrullinated histone 3 (H3Cit): preoperative and postoperative serum concentrations in each anaesthetic group (represented as median [25–75% interquartile range]). All patient groups show a decrease in H3Cit concentrations postoperatively. **B** Myeloperoxidase (MPO): preoperative and postoperative serum concentrations in each anaesthetic group (represented as median [25–75% interquartile range]). lidocaine in volatile sevoflurane or propofol intravenous anesthesia effectively reduces the postoperative increase in MPO expression. **C** Neutrophil elastase (NE): preoperative and postoperative serum concentrations in each anaesthetic group (represented as median [25–75% interquartile range]). lidocaine in volatile sevoflurane or propofol intravenous anesthesia effectively reduces the postoperative increase in NE expression. **D** matrix metalloproteinase-9 (MMP-9): preoperative and postoperative serum concentrations in each anaesthetic group (represented as median [25–75% interquartile range]). All patient groups show a increase in MMP-9 concentrations postoperatively

The reduction between preoperative and postoperative H3Cit concentrations was statistically significant in all study groups (Table 3; Fig. 2a). Postoperative concentrations of MPO (S group: 10.39[6.89–17.22] vs. 14.31[8.55–20.87] ng ml-1, *P* = 0.032; P group: 9.45[6.73–17.37] vs. 14.34[9.87–19.75] ng ml-1, *P* = 0.035) and NE (S group: 182.70[85.66-285.85] vs. 226.20[91.85-391.65] ng ml-1, *P* = 0.045; P group: 154.22[97.31–325.30] vs. 308.66[132.36-483.57] ng ml-1, *P* = 0.037) increased in non-lidocaine groups (Table 3; Fig. 2b and c), but lidocaine groups did not show significant changes postoperatively. Regrettably, this finding does not align with our initial hypothesis. Postoperative MMP-9 concentrations increased in all four groups (Fig. 2d). In summary, our study demonstrated that the addition of lidocaine effectively inhibits the increase of NETosis markers (MPO and NE) induced by breast cancer surgery.

The serum concentrations of VEGF-A after surgery showed no significant changes compared with the preoperative values in all four groups. Additionally, there were no significant differences in VEGF-A concentrations between the four groups after surgery (Table 3). Notably, in the sevoflurane groups, postoperative VEGF-A concentrations increased (S group: 103.90[40.49-192.93] vs. 114.29[55.04-170.85] ng ml-1; SL group: 90.36[34.53-170.03] vs. 95.78[50.61-202.51] ng ml-1). Conversely, in the propofol group, postoperative VEGF-A concentration decreased (P group: 100.59[62.12–136.80] vs. 96.86[52.85-129.77] ng ml-1; PL group: 123.03[82.09–189.20] vs. 117.35[89.77-190.17] ng ml-1) (Table 3).

**Table 3:** Serum biomarkers

		Group S	Group SL	Group P	Group PL	*P*
		Sevoflurane	Sevoflurane + lidocaine	Propofol-TIVA	Propofol-TIVA + lidocaine	
Trial groups		(*n* = 29)	(*n* = 30)	(*n* = 30)	(*n* = 30)	
H3Cit (ng ml^−1^)	Preoperative	4.17(3.58–4.67)	3.98(3.34–4.62)	4.05(3.44–4.52)	4.01(3.56–4.42)	0.877
	Postoperative	3.61(3.10–3.94)^*^	3.21(2.77–3.91)†	3.21(2.92–3.57)^‡^	3.08(2.66–3.65)^¥^	0.0003^*^
						< 0.0001^†^
						< 0.0001^‡^
						< 0.0001^¥^
MPO (ng ml^−1^)	Preoperative	10.39(6.89–17.22)	8.62(6.13–16.90)	9.45(6.73–17.37)	8.69(6.72–18.80)	0.928
	Postoperative	14.31(8.55–20.87)^§^	13.44(9.42–16.16)	14.34(9.87–19.75)^**^	12.08(6.37–20.23)	0.032^§^
						0.035^**^
NE (ng ml^−1^)	Preoperative	182.70(85.66-285.85)	184.70(92.66-298.65)	154.22(97.31–325.30)	172.34(102.25-300.23)	0.999
	Postoperative	226.20(91.85-391.65)^¶^	199.33(137.05–276.70)	308.66(132.36-483.57) ^##^	191.30(89.36-318.61)	0.045^¶^
						0.037^##^
MMP-9 (ng ml^−1^)	Preoperative	735.82(538.11-1036.80)	739.80(486.81-1037.14)	647.91(472.22-1268.65)	719.35(513.42-973.21)	0.999
	Postoperative	1622.04(1073.59–1945.00) ^***^	1564.15(972.44-2138.75)^ꝭ^	1364.97(868.04-1902.74)^£^	1509.65(1121.78-1946.54)^#^	< 0.0001^***^
						< 0.0001^ꝭ^
						0.013^£^
						< 0.0001^#^
VEGF-A (pg ml^−1^)	Preoperative	103.90(40.49-192.93)	90.36(34.53-170.03)	100.59(62.12–136.80)	123.03(82.09–189.20)	0.243
	Postoperative	114.29(55.04-170.85)	95.78(50.61-202.51)	96.86(52.85-129.77)	117.35(89.77-190.17)	0.234

*H3*Cit citrullinated histone H3, *MPO* Myeloperoxidase, *NE* Neutrophil elastase, *MMP *Matrix metalloproteinase, *VEGF-A *vascular endothelial growth factor-A

^*^P = 0.0003 for the preoperative vs. postoperative H3Cit concentration change in the S group

^†^*P* < 0.0001 for the preoperative vs. postoperative H3Cit concentration change in the SL group

^‡^*P* < 0.0001 for the preoperative vs. postoperative H3Cit concentration change in the *P* group

^¥^*P* < 0.0001 for the preoperative vs. postoperative H3Cit concentration change in the PL group

^§^*P* = 0.032 for the preoperative vs. postoperative MPO concentrations change in the S group

^**^P = 0.035 for the preoperative vs. postoperative MPO concentration change in the P group

^¶^*P* = 0.045 for the preoperative vs. postoperative NE concentration change in the S group

^##^*P* = 0.037 for the preoperative vs. postoperative NE concentration change in the *P* group

^***^P < 0.0001 for the preoperative vs. postoperative MMP-9 concentration change in the S group

^ꝭ^*P* < 0.0001 for the preoperative vs. postoperative MMP-9 concentration change in the SL group

^£^*P* = 0.013 for the preoperative vs. postoperative MMP-9 concentration change in the *P* group

^#^*P* < 0.0001 for the preoperative vs. postoperative MMP-9 concentration change in the PL group. All data shown are median (25–75% interquartile range)

## Discussion

This prospective, randomized, single-center trial aimed to assess the impact of anesthesia techniques and drugs on serum expression of NETosis markers (H3Cit, MPO, and NE), as well as other markers implicated in cancer dissemination (MMP-9 and VEGF-A) at 3 h post-surgery. Our study demonstrated that the addition of lidocaine effectively inhibits the increase of NETosis markers (MPO and NE) induced by breast cancer surgery.

NETosis, triggered by various stimuli like inflammation or pathogens, involves a complex process resulting in the release of neutrophil extracellular traps (NETs) [18]. Surgical stress may lead to NETosis, potentially promoting cancer metastasis. In the lidocaine group, a substantial decrease in H3Cit levels was observed post-surgery, while MPO and NE levels remained unchanged, contrasting with the control group’s significant increase in MPO and NE levels after surgery.

Our findings align with recent studies, showing a reduction in serum NETosis expression after breast cancer surgery when lidocaine was administered [19]. We propose that lidocaine’s anti-inflammatory properties or its modulation of signaling pathways may contribute to this effect, highlighting its potential in mitigating inflammation and cancer progression [7, 18, 20]. Further research is needed to elucidate the precise mechanisms involved.

Tumor angiogenesis, driven by vascular endothelial growth factor-A (VEGF-A), plays a pivotal role in tumor growth and progression [14]. Volatile anesthesia has been associated with increased tumor cell metastatic potential through up-regulating VEGF, matrix metalloproteinases (MMPs), and hypoxia-inducible factor (HIF) [5, 21, 22]. In contrast, propofol-TIVA has shown inhibitory effects on tumor cell proliferation and invasion across various cancers [23, 24]. 

Our study, despite observing lower concentrations of VEGF-A in the propofol group, did not find a statistically significant difference in VEGF-A levels. Contrary to our hypothesis, the addition of lidocaine did not significantly impact VEGF-A levels. Possible explanations include the insufficient infusion time of lidocaine or inadequate dosage for achieving a blood concentration capable of inhibiting VEGF-A. These findings align with recent in vitro evidence, utilizing a 4T1 mouse breast cancer surgical model, supporting the idea that lidocaine may not have a significant effect on angiogenic biomarkers [25]. 

It’s crucial to emphasize that the observed lower concentrations in the propofol group did not translate into a statistically significant difference. Further exploration is needed to understand the complex interactions influencing VEGF-A levels in the context of different anesthesia regimens.

Metastasis initiation involves cancer cells undergoing a loss of intercellular adhesion through the modification of cell surface proteins during the epithelial-mesenchymal transition (EMT). This process is followed by the facilitated degradation of the extracellular matrix, mediated by matrix metalloproteinases and urokinase plasminogen activators [19]. MMP-9, a key player in the EMT process and extracellular matrix regulation, has been implicated in the metastatic cascade [26]. 

Our study observed an increase in MMP-9 levels following surgery across all groups. Interestingly, the rise in MMP-9 levels was comparatively modest in the propofol group. However, the administration of lidocaine infusion did not show any association with the elevation of MMP-9 levels after surgery. This unexpected finding challenges our initial anticipation that the inclusion of lidocaine would mitigate the surge in MMP-9 levels. These results align with the in vitro findings of Wall TP et al., who reported that intravenous lidocaine failed to diminish MMP-9 levels in a surgical model of mouse breast cancer [27]. Further exploration is needed to unravel the intricate interactions influencing MMP-9 dynamics in the context of different anesthesia regimens.

Each of the four study groups received comparable intraoperative opioid doses, although the exact amounts are not specified in the current text. Existing literature suggests a potential link between opioids and tumor metastasis and recurrence, but evidence from retrospective human data remains inconclusive. Trials advocating regional anesthesia techniques, involving opioid retention, have demonstrated advantages in surgical populations, yet the origin of these benefits—whether directly from opioid avoidance or supplementary advantages of regional anesthesia—remains uncertain.

Controversy surrounds the role of opioids in the literature, with recent research indicating that, for brief periods of exposure, opioids may not exert a substantial clinical impact on the growth and recurrence rates of tumor cells [28]. Nevertheless, until robust prospective randomized trials in humans are completed, opioids continue to play a pivotal role in perioperative analgesia. Further investigation is warranted to discern the nuanced relationship between opioids and cancer outcomes.

Several limitations warrant acknowledgment in our study. Firstly, logistical and personnel constraints prevented the extension of lidocaine administration into the postoperative recovery phase, which would have been ideal. We did not measure plasma concentrations of lidocaine, though no adverse effects, such as ECG changes, were observed. Additionally, lidocaine concentrations in primary tissue tumors were not assessed, and the direct impact of lidocaine on cancer cells or specimens was not directly evaluated, leaving assumptions regarding the correlation between reductions in NETosis and angiogenesis markers and improved patient outcomes. Long-term follow-up data absence further hinders substantiating these claims.

## Conclusions

In conclusion, our study shows that incorporating lidocaine in volatile sevoflurane or propofol intravenous anesthesia effectively reduces the postoperative increase in NETosis (NE and MPO), a biomarker associated with metastasis risk. However, to assess the potential impact on cancer recurrence, large-scale, multi-center, and long-term follow-up clinical trials are necessary. These trials will provide a more comprehensive understanding of the implications of lidocaine in general anesthesia and its role in cancer outcomes.

## Data Availability

All the data used and analyzed are available from corresponding authors upon the reasonable request.

## Abbreviations

NETosis: Neutrophil extracellular trapping
VEGF-A: Vascular endothelial growth factor A
H3Cit: Citrullinated histone H3
MPO: Myeloperoxidase
NE: Neutrophil elastase
MMPs: Matrix metalloproteinases
MMP-9: Matrix metalloproteinase-9
TIVA: Propofol-based total intravenous anesthesia
NETs: Neutrophil extracellular traps
ASA: American Society of Anesthesiology
HRP: Horseradish peroxidase
SABC: Streptavidin-Biotin Complex
TMB: Tetramethylbenzidine
TNM: Tumor-Node-Metastasis
HIF: Hypoxia-inducible factor
EMT: Epithelial-mesenchymal transition
ECG: Electrocardiogram
BMI: Body Mass Index
NACT: New Adjuvant Chemotherapy Treatment
Tis: Tumor in situ
NST: No special type
HER2: Human epidermal growth factor receptor 2
HR: Heart rate
MAP: Mean arterial pressure
